# Studying the Effect of Long COVID-19 Infection on Sleep Quality Using Wearable Health Devices: Observational Study

**DOI:** 10.2196/38000

**Published:** 2022-07-05

**Authors:** Mario Mekhael, Chan Ho Lim, Abdel Hadi El Hajjar, Charbel Noujaim, Christopher Pottle, Noor Makan, Lilas Dagher, Yichi Zhang, Nour Chouman, Dan L Li, Tarek Ayoub, Nassir Marrouche

**Affiliations:** 1 Tulane University School of Medicine New Orleans, LA United States; 2 Department of Medicine Emory University School of Medicine Atlanta, GA United States

**Keywords:** COVID-19, digital health, wearables, sleep, long COVID-19, wearable device, demographic, biometric, patient data, sleep architecture, health data, health monitoring

## Abstract

**Background:**

Patients with COVID-19 have increased sleep disturbances and decreased sleep quality during and after the infection. The current published literature focuses mainly on qualitative analyses based on surveys and subjective measurements rather than quantitative data.

**Objective:**

In this paper, we assessed the long-term effects of COVID-19 through sleep patterns from continuous signals collected via wearable wristbands.

**Methods:**

Patients with a history of COVID-19 were compared to a control arm of individuals who never had COVID-19. Baseline demographics were collected for each subject. Linear correlations among the mean duration of each sleep phase and the mean daily biometrics were performed. The average duration for each subject’s total sleep time and sleep phases per night was calculated and compared between the 2 groups.

**Results:**

This study includes 122 patients with COVID-19 and 588 controls (N=710). Total sleep time was positively correlated with respiratory rate (RR) and oxygen saturation (SpO_2_). Increased awake sleep phase was correlated with increased heart rate, decreased RR, heart rate variability (HRV), and SpO_2_. Increased light sleep time was correlated with increased RR and SpO_2_ in the group with COVID-19. Deep sleep duration was correlated with decreased heart rate as well as increased RR and SpO_2_. When comparing different sleep phases, patients with long COVID-19 had decreased light sleep (244, SD 67 vs 258, SD 67; *P*=.003) and decreased deep sleep time (123, SD 66 vs 128, SD 58; *P*=.02).

**Conclusions:**

Regardless of the demographic background and symptom levels, patients with a history of COVID-19 infection demonstrated altered sleep architecture when compared to matched controls. The sleep of patients with COVID-19 was characterized by decreased total sleep and deep sleep.

## Introduction

Although COVID-19 is primarily known as a pulmonary disease [1], literature suggests significant consequences regarding daily activities and mental health due to the infection itself or associated quarantine [2]. Moreover, reports indicate increased incidences of psychologic and psychiatric conditions during the pandemic such as sleep disturbances and decreased accessibility to health care [3,4]. Thus, there is a need for remote continuous monitoring, telemedicine, and digital health monitoring systems to bridge the gap between patients and physicians [5].

The sleep cycle is traditionally divided in two phases: rapid eye movement (REM) sleep and nonrapid eye movement (NREM). Furthermore, NREM sleep is divided into the three subphases of (1) awake, (2) light, and (3) deep sleep. Human body usually cycles through these phases 4 to 6 times per night with 90 minutes in each stage [6]. Initially, sleep has been studied using polysomnography, which is a multisensor system that has been the gold standard for analyzing sleep stages and sleep-related disorders [7]. However, polysomnography has many drawbacks, such as the need for a hospital stay and its high-cost logistics such as the use of complex hardware needed for electroencephalographic, electromyographic, and electrooculographic assessments. All those factors can alter physiological sleep architecture and bias the results. Consequently, less than half of sleep studies nowadays are conducted in formal sleep facilities [8]. Having said that, wearable technology has been developed in the last decade, which consists of smart devices or gadgets worn close to or in contact with the skin used to capture biometric data [9]. With the recent trend of wearables, we have seen the development of photoplethysmography (PPG) technology to analyze different sleep phases, avoiding challenges that accompany the traditional polysomnography exam. In fact, reflective light emitted by the wearable device allows to measure blood volume changes in the vessels, which allows for the accurate measurement of heart rate (HR) and heart rate variability (HRV) [10]. HRV serves as a surrogate to estimate the effect of both sympathetic and parasympathetic nervous systems on the cardiovascular system. In addition, activities of both parasympathetic and sympathetic nervous systems vary in different sleep phases. For example, increased parasympathetic nervous system activity and therefore decreased HR was noticed in deeper stages of sleep [11,12]. Consequently, machine learning algorithms have been developed using the relationship between biometrics (such as HR and HRV) and sleep cycle to define sleep phases using PPG [13-16].

Long COVID-19 syndrome is defined as symptoms that persist after acute COVID-19 infection; however, the definitions vary by literature [17-19]. Previous studies have shown increased sleep disturbances and decreased sleep quality during and after COVID-19 infection [20,21]. However, those studies focused on qualitative analyses based on subjective measurements and survey responses rather than quantitative data [22,23]. Hence, in this paper, we study and evaluate the long-term effects of COVID-19 on sleep patterns using the continuously monitored metrics from wristband devices.

## Methods

### Study Design

Wearables to Investigate the Long Term Cardiovascular and Behavioral Impacts of COVID-19 (WEAICOR) is a prospective observational study of subjects 18 years or older who were monitored using the Biostrap wearable or wristband device. The study aims to identify the impact of long COVID-19 infection on sleep using wearables. In this analysis, we sought to compare continuous data recorded using a wearable device between patients who were diagnosed and recovered from COVID-19 and controls who were never diagnosed with the disease.

**Figure 1:**
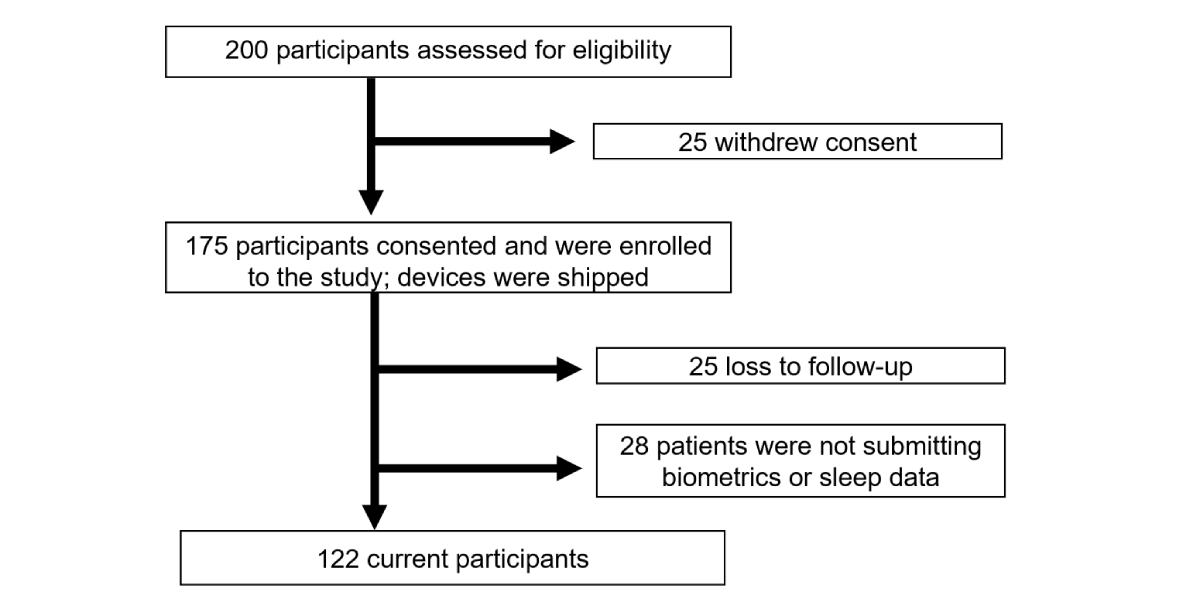
Study flowchart.

Patient’s enrollment flowchart is represented in Figure 1. After eligibility screening and signing electronic consent forms, all subjects were sent a Biostrap device by mail to continuously monitor their biometric data. Biometric parameters included HR, HRV, respiratory rate (RR), and oxygen saturation (SpO_2_). Device Instructions tailored to the study were provided by phone call by the study coordinator, along with a recorded video detailing the steps to activate the device with the mobile app.

### Ethics Approval

WEAICOR study was approved by Tulane University Institution Review Board on June 09, 2020 (Study #2020-678).

### Study Population

In this analysis, patients who had COVID-19 and recovered (study arm) will be compared to a control arm of participants who never had COVID-19 or associated symptoms. The study arm recruitment was carried out through flyers and advertisements on different platforms of social media along with mass emails generated to the Tulane staff and student body. A total of 200 participants were assessed for eligibility by September 2021. The control data were collected from a group of participants who opted in to an internal Biostrap study from April 12, 202, to July 31, 2020, as a part of their COVID-19 initiative. The participants received a baseline questionnaire collecting demographic and medical history data. Additionally, a daily survey was sent to all individuals to identify any COVID-19 symptom or positive COVID-19 case in each participant’s household. Only individuals who consistently answered “No” regarding a positive COVID-19 diagnosis and denied related symptoms were included in the control group. Additionally, the existing users were willing to contribute their deidentified data for research. We secured informed consent forms and listed Tulane University as an organization with data access.

### Biostrap Device

**Figure 2:**
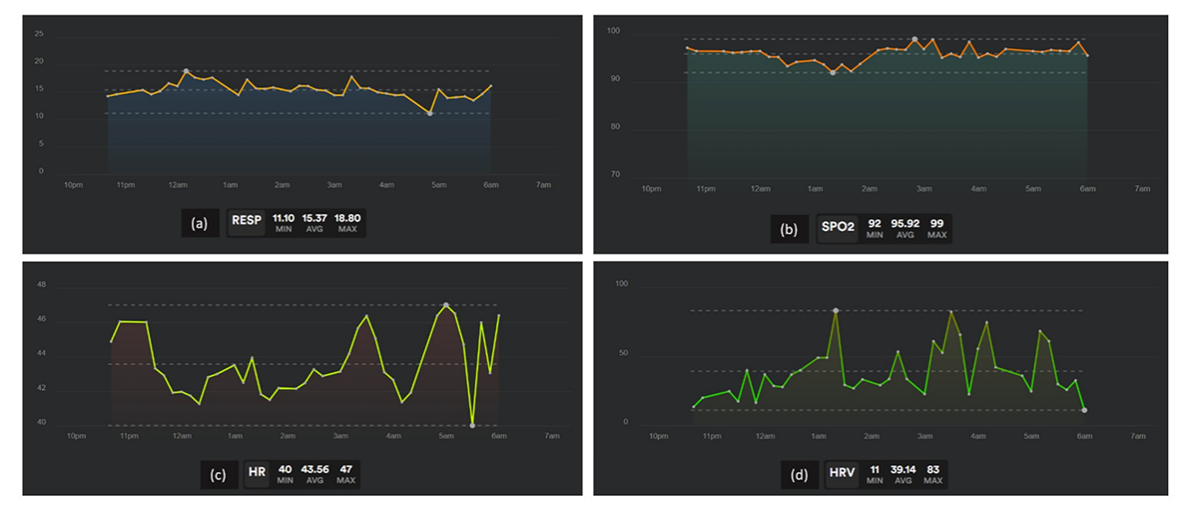
Recording example of biometrics during a night for a patient with long COVID-19. (a) RESP: respiratory rate (respirations per minute); (b) SpO_2_: saturation of oxygen (%); (c) HR: heart rate (beats per minute); and (d) HRV: heart rate variability (beats per minute).

**Figure 3:**
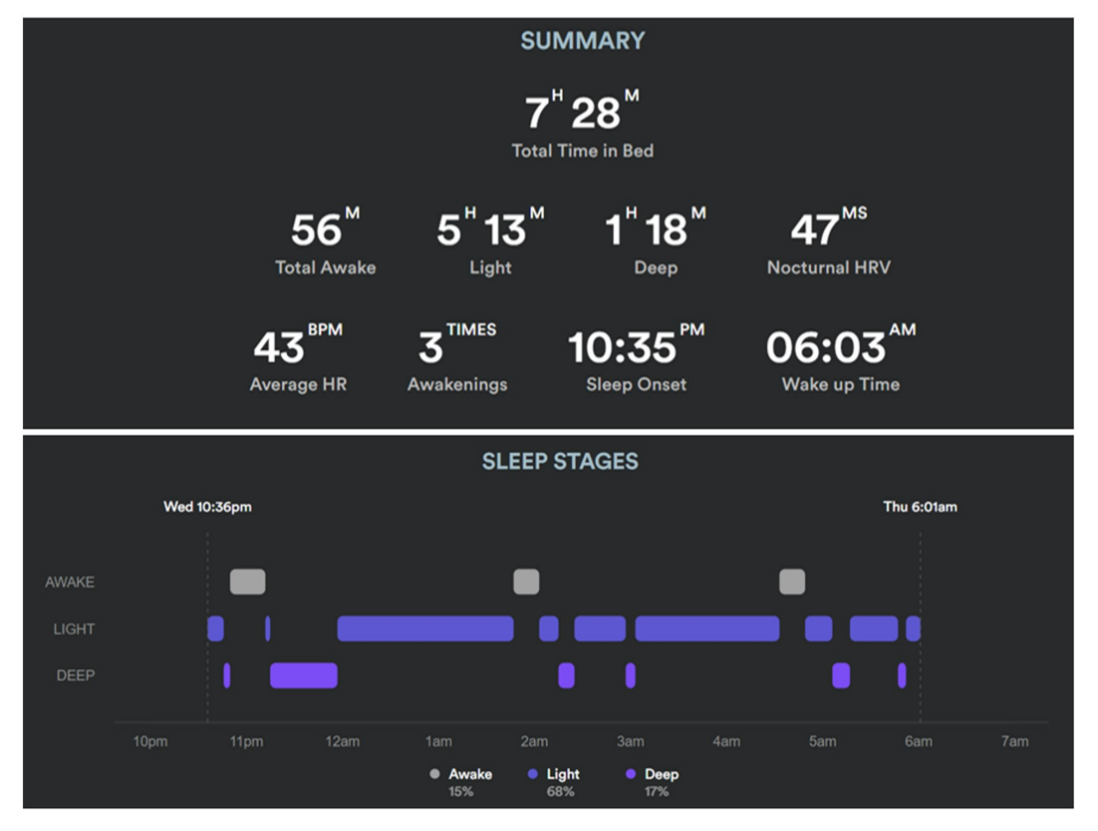
Recording example of sleep summary and sleep phases during a night for a patient with long COVID-19. HR: heart rate; HRV: heart rate variability.

Biostrap is a PPG-based smartband that records patients’ vitals at rest with 5-minute intervals and generates graphic results and reports on the Biostrap mobile app. PPG is an optical technique for detecting blood volume changes within the blood vessels by the changes in the light received from the photodiode to estimate physiological parameters. Biometrics such as HR, HRV, RR, and SpO_2_ along with others related to the cardiovascular and autonomic nervous systems can be computed noninvasively using collected infrared signals. When paired with infrared signals, a red-light signal enables SpO_2_ estimation. The combination of all those parameters along with arm movement enables us to classify sleep cycle into the 3 different phases of awake sleep, light sleep, and deep sleep. Example of biometric recordings (Figure 2) and sleep analysis recordings (Figure 3) are provided for simplification. Figure 2 shows biometrics recordings during a single night for a patient with COVID-19. Figure 3 describes the summary report and time spent in different sleep phases in a single night for a patient with COVID-19. PPG and accelerometer data collected from the wrist are transferred by the mobile app to the Biostrap cloud server, where they undergo signal processing and machine learning algorithms to generate physiological data at rest and transfer it to Tulane’s data warehouse server. The accuracy and reproducibility of the Biostrap device in assessing basic physiological data have already been reported in previously published studies [24,25].

### Patient Follow-up

The research nurse and coordinator assigned to the study used Biostrap’s remote data to ensure adequate data generation, patient compliance, and technical troubleshooting. Throughout the study, they actively followed up with the patients through phone calls and emails to address any problem or concern.

### Data Analysis

The following baseline characteristics were collected for each participant: age, gender, BMI, comorbidities, educational level, and COVID-19 symptoms severity level. The means of these baseline characteristics were calculated for COVID-19 and controls and compared using 2-sample *t* test (2-tailed).

**Figure 4:**
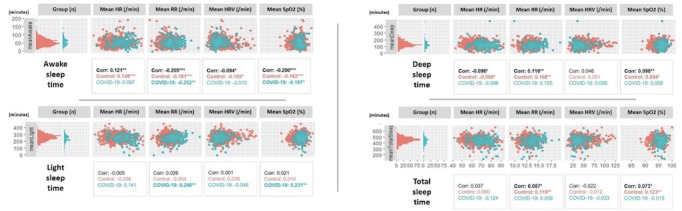
Correlations between different sleep phases and biometrics. Corr: correlation with the whole cohort; HR: heart rate; HRV: heart rate variability; RR: respiratory rate; SpO_2_: oxygen saturation. *P<.05; **P<.01; ***P<.001.

For each participant, the average duration in different sleep phases per night (awake, light, deep, and total) was calculated. For each sleep phase, we took the number of minutes per phase per day and calculated the average over the total number of days. Pearson bivariate linear correlations among the mean duration in sleep phases and means of biometrics (HR, HRV, RR, and SpO_2_) were performed for COVID-19, controls, and the whole study population (specified as “Cohort” in the results section) to evaluate the association between the different components of the autonomic system and sleep cycle (Figure 4). The direction of changes in biometrics and duration of sleep phases will allow us to understand more the interaction between these 2 systems.

In addition, the participants from the whole cohort were divided into groups depending on their biometrics average during sleep (higher HR: >80 beats per minute vs lower HR: <80 beats per minute [26]; higher HRV: >20 milliseconds vs lower HRV: <20 milliseconds; and higher RR: >20 breaths per minute vs lower RR: <20 breaths per minute). Sleep phases (total, awake, light, and deep sleep) between the different groups were compared using Mann-Whitney *U* test. Patients with low HRV (n=27) and high RR (n=0) were little in number, and therefore the analysis was not statically significant for HRV and was not feasible for RR.

The mean measurements for each participant’s total sleep time and sleep phases per night (awake, light, deep, and total) were calculated and weighted proportionally to the number of days each participant submitted data. For example, a participant with 25 nights of sleep data would have half of the weight of one with 50 nights of sleep data. From that weighted set, the median of each group’s sleep times was taken and recorded, as the distributions of mean sleep times across both groups departed significantly from normality according to the Shapiro Wilk test. Distributions of sleep times in the control group and the group with COVID-19 were compared using the 2-Sample Wilcoxon (Mann-Whitney *U*) test. The same analysis was conducted in an unweighted manner, where, for example, a subject with 25 nights of sleep data had just as much an effect on the test as a subject with 50 nights of sleep data. Two-sided *P* values of less than .05 were considered significant.

To mitigate potential selection bias arising from the observational nonrandomized study design, we applied propensity score matching and achieved a more balanced control group. During the matching process, the study participants with a history of COVID-19 were matched 1:1 to the participants without any history of COVID-19 by calculating the propensity score of the participants having COVID-19. The propensity score was carried out by estimating the probability of each participant having a history of COVID-19 based on age, BMI, and gender through multivariable logistic classifier.

## Results

### Baseline Characteristics

We included 122 patients in the group with COVID-19 and 588 participants in the control group. Patients in the COVID-19 arm were younger than controls (of 42.8, SD 15.5 vs 46.0, SD 14.0 years; *P*=.02). Patients with COVID-19 were 32% (39/122) female, and the controls were 22% (129/588) female (*P*=.33). There were no other significant differences in baseline characteristics and comorbidities between the 2 arms. Notably, both populations tended to be young and healthy, with most participants having few or no comorbidities. In the group with COVID-19, most of the patients considered (n=112, 92%) were not hospitalized during their COVID-19 diagnosis. Data were collected 171 (SD 114) days after their COVID-19 diagnosis. All baseline characteristics for both COVID-19 and control groups are represented in Table 1.

**Table 1:** Baseline demographic and clinical characteristics of COVID-19 and control arms.

Characteristics			COVID-19 (n=122)		Control (n=588)		*P* value
Age (years) mean (SD)			41.32 (15.7)		45.99 (14.0)		.001
**Gender, n (%)**							.33
	Male	76 (62)		453 (77)			
	Female	46 (38)		135 (23)			
BMI (kg/m^2^)			28.7 (8.6)		27.1 (5.7)		.001
**Race or ethnicity, n (%)**							.36
	White	71 (58)		465 (79)			
	African American or Black	20 (16.5)		3 (0.5)			
	Asian	12 (10)		29 (5)			
	Latino or Hispanic	5 (4.5)		41 (7)			
	Others	13 (11)		50 (8.5)			
**Comorbidity, n (%)**							.96
	None	88 (72)		506 (86)			
	Diabetes	6 (5)		12 (2)			
	Immune system deficiencies or HIV	1 (1)		12 (2)			
	Heart conditions	4 (3)		12 (2)			
	Asthma or chronic lung disease	15 (12)		24 (4)			
	Extreme obesity	5 (4)		18 (3)			
	Cancer treatment	4 (3)		6 (1)			
**Education level, n (%)**							.21
	Bachelor’s degree	27 (22)		247 (42)			
	Some college	30 (24)		65 (11)			
	Associate degree	16 (13)		41 (7)			
	Master’s degree	28 (23)		112 (19)			
	Doctorate	1 (1)		35 (6)			
	Professional	10 (8)		59 (10)			
	Others	11 (9)		29 (5)			

### Average Follow-up of the 2 Groups Using the Biostrap Device

Controls were followed up for 64 (SD 28) days and patients with long COVID-19 were followed up for 55 (SD 66) days. For the weighted analysis, 37,709 recorded days (103.2 years) were collected for the control group and 7228 recorded days (19.8 years) were collected for patients with COVID-19.

### Correlations Between Biometrics and the Different Phases of the Sleeping Cycle

All the correlations between sleep phases and biometrics are summarized in Figure 4.

#### Total Sleep Cycle

Total sleep time was correlated with RR (*r*=0.084, *P*≤.05 for cohort and *r*=0.119, *P*≤.01 for controls) and SpO_2_ (*r*=0.076, *P*≤.05 for cohort and *r*=0.123, *P*≤.01 for controls). Total sleep time was not significantly correlated with HR (*P*>.05) and HRV (*P*>.05).

#### Awake Sleep Phase

Significant correlations were found between HR (*r*=0.109, *P*≤.01 for cohort and *r*=0.148, *P*<.001 for controls), RR (*r*=–0.201, *P*<.001 for cohort and *r*=–0.161, *P*<.001 for controls), HRV (*r*=–0.099, *P*≤.01 for cohort and *r*=–0.105, *P*≤.05 for controls), SpO_2_ (*r*=–0.205, *P*<.001 for cohort and *r*=–0.162, *P*<.001 for controls), and awake sleep phase.

#### Light Sleep Phase

For light sleep phase, only RR (*r*=0.358, *P*<.001) and SpO_2_ (*r*=0.249, *P*<.001) in the COVID-19 group were found to be correlated with the time spent in this phase. There was no significant correlation between light sleep and HR nor between light sleep and HRV (*P*>.05).

#### Deep Sleep Phase

As for deep sleep, the time spent in this phase was correlated with HR (*r*=–0.093, *P*≤.05 for cohort and *r*=–0.098, *P*≤.05 for controls), RR (*r*=0.121, *P*≤.01 for cohort and *r*=0.108, *P*≤.01 for controls), and SpO_2_ (*r*=0.106, *P*<.001 for cohort and *r*=0.094, *P*≤.01 for controls). However, it did not significantly correlate with HRV (*P*>.05). As seen in Figure 4, awake sleep significantly correlates with HR in all participants (*r*=0.121, *P*<.01), in the control group (*r*=0.148, *P*<.001), but not in patients with COVID-19 (*r*=0.097, *P*>.05); awake sleep also significantly correlates with RR in all participants (*r*=–0.205, *P*<.001), in the control group (*r*=–0.161, *P*<.001), and in patients with COVID-19 (*r*=–0.252, *P*<.01); awake sleep also correlates with HRV in all participants (*r*=–0.094, *P*<.05), in the control group (*r*=–0.094, *P*<.05), but not in patients with COVID-19 (*r*=–0.010, *P*>.05); awake sleep correlates with SpO_2_ in all participants (*r*=–0.200, *P*<.001), in the control group (*r*=–0.162, *P*<.001), and in patients with COVID-19 (*r*=–0.187, *P*<.05). The same interpretation can be drawn from Figure 4 for light sleep, deep sleep, and total sleep.

### Comparison of Sleep Cycle in Patients With Lower vs Higher HR

**Figure 5:**
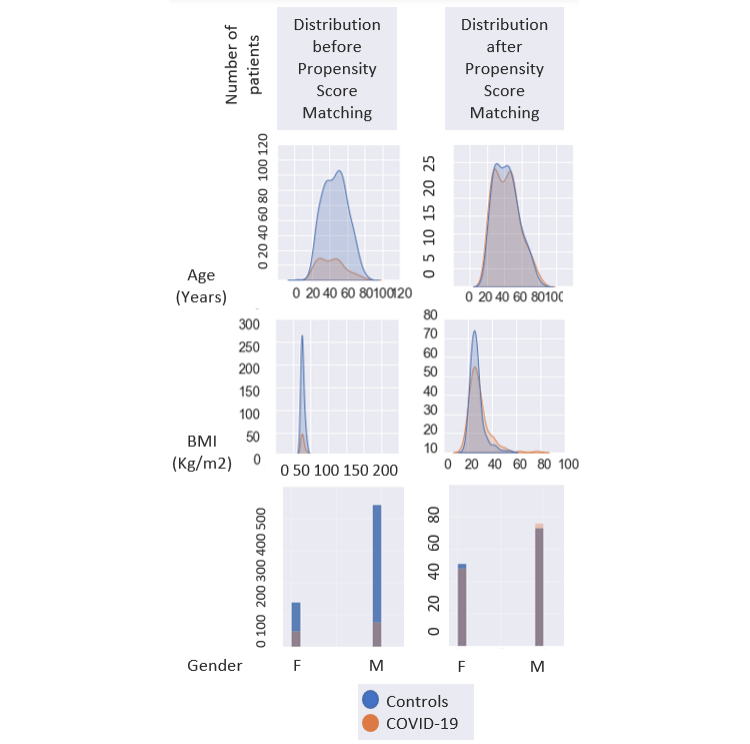
Summary representation of propensity score matching for age, BMI, and gender. F: Female; M: Male. BMI: body mass index.

After dividing the cohort into patients with higher HR (50/710 patients, 7%) and lower HR (660/710 patients, 93%), patients with higher HR had more time in awake sleep (65 minutes vs 55 minutes, *P*=.02) and less time in light (232 minutes vs 258 minutes, *P=*.001), deep (128 minutes vs 135 minutes, *P*=.1), and total sleep (425 minutes vs 449 minutes, *P*=.006; Figure 5). The number of patients and the different results are listed in Table 2.

**Table 2:** Different biometric groups with respective number of patients.

Group	Number of patients	Awake sleep phase (min)^a^	Light sleep phase (min)^b^	Deep sleep phase (min)^c^		Total sleep phase (min)^d^
Higher HR^e^ (>80 beats/min)	50	65	232	128		425
Lower HR (<80 beats/min)	660	55	258	135		449
Higher RR^f^ (>20 respirations per minute)	0	—^g^	—	—	—	
Lower RR (<20 respirations per minute)	710	—	—	—	—	
Higher HRV^h^ (>20ms)	683	—	—	—	—	
Lower HRV (<20ms)	27	—	—	—	—	

^a^*P*=.02.

^b^*P*=.001.

^c^*P*=.1.

^d^*P*=.006.

^e^HR: heart rate.

^f^RR: respiratory rate.

^g^Statistical analysis was not performed to assess the differences between these groups because of the low number of patients in Higher RR and Lower HRV groups.

^h^HRV: heart rate variability.

### Comparison of Sleep Length Between Patients With COVID-19 and Controls

#### Unweighted Analysis

In the unweighted analysis, patients with long COVID-19 had less total sleep time when compared to controls (433, SD 85 vs 450, SD 68; *P*<.001).

#### Weighted Analysis

In the weighted analysis, patients with long COVID-19 had statistically but not clinically significant increased total sleep time when compared to control group (451.4, SD 65 vs 451.7, SD 87 minutes, *P*<.001).

#### Propensity Match Analysis

After performing a propensity match analysis, 122 patients with COVID-19 were compared to 122 matched controls. Total sleep time was found to be decreased in the group with COVID-19 compared to controls (433, SD 85 vs 450, SD 68; *P*=.004). A schematic representation of the data distribution before and after propensity score matching was shown for better visualization (Figure 5).

### Comparison of Sleep Cycle Phases Between Patients With COVID-19 and Controls

#### Unweighted Analysis

**Figure 6:**
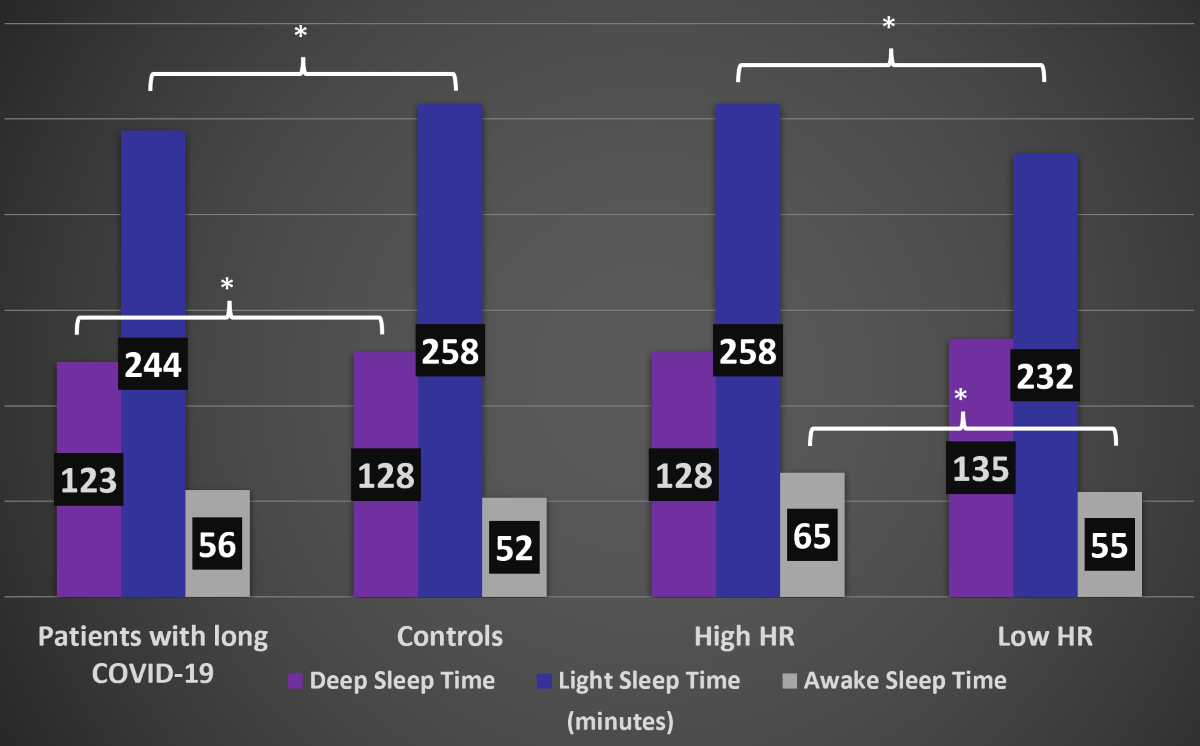
Difference in weighted sleep phases between different groups. High heart rate (HR): >80 beats per minute. Low HR: <80 beats per minute; *P<.05.

In the unweighted analysis, there was no statistical awake sleep time difference between the 2 groups (52, SD 32 for controls vs 56, SD 31 for cohort; *P*=.4). However, patients with long COVID-19 had decreased light sleep (244, SD 67 vs 258, SD 67; *P*=.003) and decreased deep sleep (123, SD 66 vs 128, SD 58; *P*=.02; Figure 6).

#### Weighted Analysis

When comparing weighted different sleep phases, patients with long COVID-19 had increased awake sleep time (62, SD 25 vs 52, SD 32; *P*<.001) decreased light sleep time (251, SD 82 vs 260, SD 64; *P*<.001), and decreased deep sleep (126, SD 71 vs 131, SD 59; *P*<.001).

#### Propensity Score Matching

After matching the 2 groups for age, sex, and BMI, patients with COVID-19 have decreased deep sleep when compared to controls (123, SD 66 vs 128, SD 57; *P*<.004). However, the differences in light sleep (244, SD 67 vs 259, SD 67; *P*=.39) and awake sleep (56, SD 31 vs 57, SD 32; *P*=.71) were no longer significant.

## Discussion

### Overview

In our study, we report 2 major findings. First, increased total sleep time and time spent in deep sleep were associated with increased RR and SpO_2_, and decreased HR in the full cohort. Second, the group with long COVID-19 had altered sleep architecture characterized by decreased total and deep sleep times when compared to matched controls.

### Association Between Biometrics and Sleep Phases

Decreased oxygen saturation during sleep can be due to different pathologies and has the potential to inflict significant negative physiological and psychological consequences [27]. In our cohort, decreased RR and SpO_2_ were associated with increased time in awake sleep phase. Moreover, increased RR and SpO_2_ were associated with increased total sleep time and deep sleep. Our findings suggest difficulty transitioning into deep sleep of patients with decreased respiratory function and thus the need for good oxygenation and respiratory function to maintain a physiological sleep cycle. This is in line with the increased sleeping disturbances noticed in patients with severe COVID-19. Huang et al [28] showed that the risk of severe infection was 6 to 8 times more associated with decreased sleep status and reduced sleeping hours [28]. Additionally, the reduction in average daily sleep time significantly increased the likelihood of infection severity, stressing on the intertwined relationship between sleep and respiratory function [28]. However, the results reported in this study were extracted from self-reported questionnaires in comparison to our quantitative results. Moreover, increased RR and SpO_2_ in the group with COVID-19 was correlated with increased time spent in light sleep. Light sleep, which is one of the different phases of NREM sleep, was found to have an important role in memory formation and consolidation as well as in motor skill speed and performance [29,30]. Therefore, the association of optimal respiratory function during sleep and improved sleep quality may improve activities of daily living and quality of life in addition to immunity and response to infections.

As for the autonomous system, increased HR and decreased HRV were correlated with increased time in awake phase, whereas decreased HR was correlated with increased time in deep sleep. Increased HR during sleep was associated with increased cardiovascular comorbidities [31]. This was widely studied in night-shift workers, who had misalignment between the endogenous circadian clock and the sleep schedule, leading to increased cardiovascular events [32]. Our data showing that participants with increased HR have increased time in awake sleep and less time in deep sleep might indicate a difficulty in transitioning from light to deep sleep among patients with increased HR. Disturbances in the configuration of these 2 systems may lead to adverse repercussions and clinical outcomes.

The key benefit of continuous monitoring with wearables lies in the capability to detect these vulnerable populations who may have early sleep or biometric disturbances. The collection of real-time data from wearables can allow the physician to manage patients at a very early stage. By combining data from biometrics and sleep phases, physicians will be able to have an overview on patients’ autonomic system activity. These findings, sometimes subclinical, will be useful as a significant decision support tool for physicians to employ preventative and personalized medicine even before diagnosing the problem.

### Long COVID-19’s Effect on Sleep

Previous studies have shown increased psychological disturbances in addition to the physical component associated with COVID-19 infection [22,33]. In fact, decreased sleep quality and insomnia problems have been reported during the pandemic. However, most sleep-related studies focused primarily on health care workers rather than the general population [34,35]. For example, Zhang et al [35] found that almost one-third of health care workers had insomnia symptoms during the pandemic, and that the related factors included education level, isolation environment, and psychological stressors [35]. However, most of these studies were qualitative and survey-based rather than quantitative [22,23]. Thus, there was a need for a quantitative approach to assess long COVID-19’s effect on sleep.

In our study, participants in the group with long COVID-19 had increased awake sleep time and decreased light and deep sleep time. During sleep, the body secures restorative functions related to immunity [36], the cardiovascular system [37], and metabolic functions [38]. Alterations in non-REM sleep phases may therefore predispose health-related problems. In addition, altered sleep architecture was shown to increase stress levels by increasing stress hormones [39-42]. These findings especially after recovering from the infection support the fact that COVID-19 may present with long-standing symptoms such as autonomic and neurologic disturbances. This is in alignment with what is called “Long COVID-19” syndrome or “COVID-19 Brain fog,” which is characterized by fatigue, difficulty concentrating, and sleep disorders even after the acute infection [43].

### Limitations

Our study has several limitations. First, this is a single-center study, limiting the reproducibility of the results among a wider population. Second, baseline data regarding physiological state of participants with COVID-19 is not available as they did not have the device before COVID-19 infection. Third, the PPG technology used was not developed to accurately characterize REM sleep, and thus, REM sleep has been omitted from the analysis. Finally, the controls were recruited based on a patient-reported survey, and therefore they might have had COVID-19 without knowing.

### Conclusion

Study participants with improved cardiovascular and respiratory functions had better sleep architecture. Moreover, patients who were diagnosed with COVID-19, including young and healthy patients, demonstrated altered sleep architecture when compared to matched controls. The sleeping data of patients with COVID-19 were characterized by decreased total sleep and deep sleep times. Future studies should evaluate the physical and psychological impact of sleep disturbance among patients with long COVID-19.

## Abbreviations

HR: heart rate
HRV: heart rate variability
NREM: nonrapid eye movement
PPG: photoplethysmography
REM: rapid eye movement
RR: respiratory rate
SpO_2_: oxygen saturation
WEAICOR: Wearables to Investigate the Long Term Cardiovascular and Behavioral Impacts of COVID-19

## Data Availability

The data that support the findings of this study are available from the corresponding author, N Marrouche, upon reasonable request.
